# Supplementary figures and images for: Identification of a metabolic-related gene signature predicting the overall survival for patients with stomach adenocarcinoma
Source: PeerJ. 2021 Feb 8;9:e10908. doi: 10.7717/peerj.10908 (PMC7877239; doi:10.7717/peerj.10908)

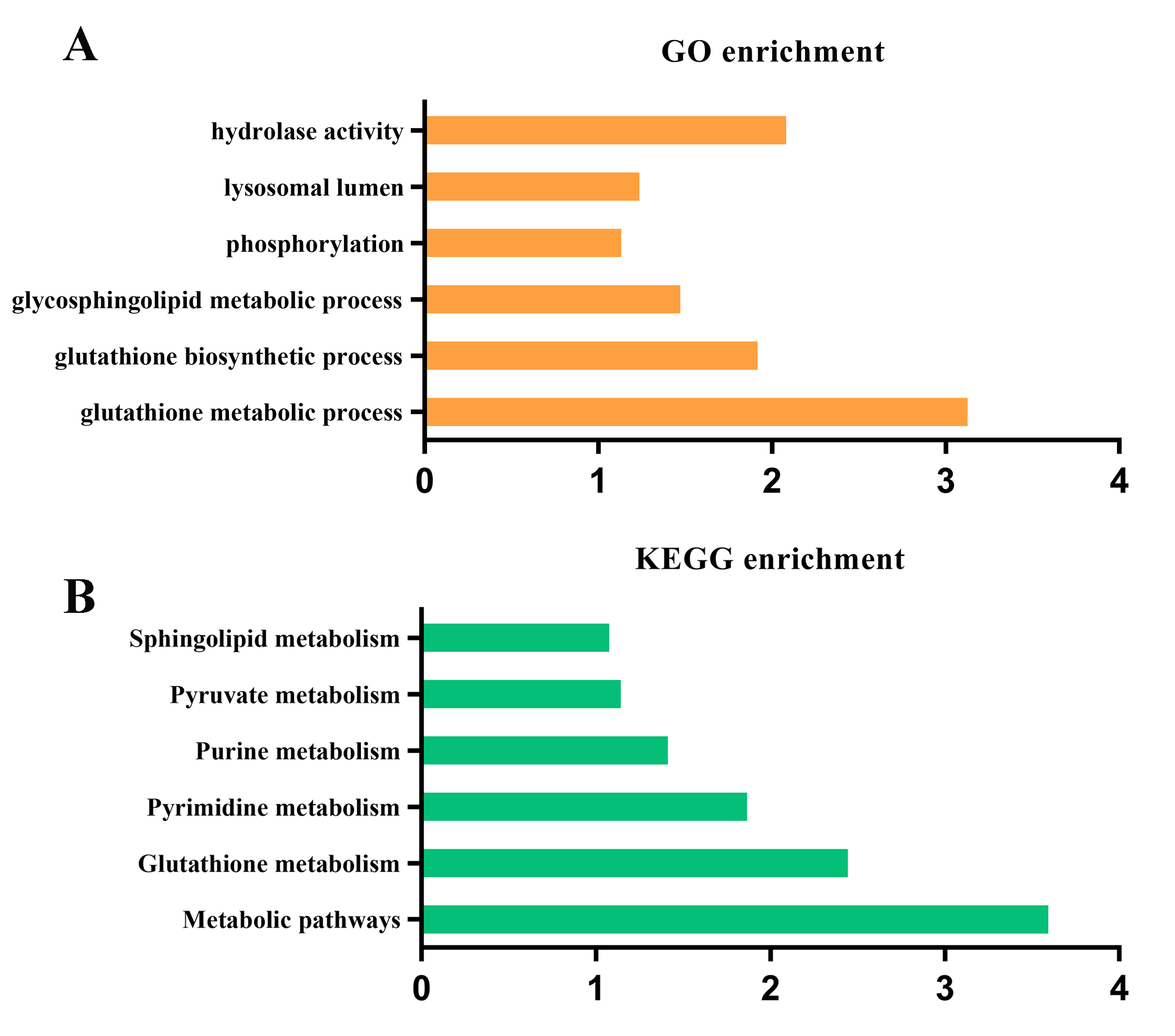

Supplement: Supplemental Information 3 [file peerj-09-10908-s003.png]
